# Mechanisms underlying interactions between two abundant oral commensal bacteria

**DOI:** 10.1038/s41396-021-01141-3

**Published:** 2021-11-03

**Authors:** Dasith Perera, Anthony McLean, Viviana Morillo-López, Kaileigh Cloutier-Leblanc, Eric Almeida, Kiana Cabana, Jessica Mark Welch, Matthew Ramsey

**Affiliations:** 1 The University of Rhode Island, Kingston, RI 02881 USA; 2 Marine Biological Laboratory, Woods Hole, MA 02543 USA

**Keywords:** Microbiome, Bacterial physiology, Bacteriology, Biofilms, Microbial ecology

## Abstract

Complex polymicrobial biofilm communities are abundant in nature particularly in the human oral cavity where their composition and fitness can affect health. While the study of these communities during disease is essential and prevalent, little is known about interactions within the healthy plaque community. Here we describe interactions between two of the most abundant species in this healthy microbiome, *Haemophilus parainfluenzae* and *Streptococcus mitis*. We discovered that *H. parainfluenzae* typically exists adjacent to mitis group streptococci in vivo with which it is also positively correlated based on microbiome data. By comparing in vitro coculture data to ex vivo microscopy we revealed that this co-occurrence is density dependent and further influenced by H_2_O_2_ production. We discovered that *H. parainfluenzae* utilizes a more redundant, multifactorial response to H_2_O_2_ than related microorganisms and that this system’s integrity enhances streptococcal fitness. Our results indicate that mitis group streptococci are likely the in vivo source of NAD for *H. parainfluenzae* and also evoke patterns of carbon utilization in vitro for *H. parainfluenzae* similar to those observed in vivo. Our findings describe mechanistic interactions between two of the most abundant and prevalent members of healthy supragingival plaque that contribute to their in vivo survival.

## Introduction

Taxon-taxon interactions are of great importance for understanding the structure and function of natural biofilms. Many bacteria occur in nature as part of complex polymicrobial biofilms [1]. The behavior of individual species within these biofilms is elaborate and likely influenced by the spatial organization between physical and chemical substrates [1, 2] produced by distinct species [3–5]. Metagenomic and metatranscriptome techniques give us an unprecedented ability to observe group composition and behaviors, but cannot resolve which species might be influencing one another nor describe their spatial interactions. Metabolite-based interactions can influence community composition through crossfeeding or trophic interactions [6, 7], complementation of auxotrophies [8] as well as competition for nutrients and production of inhibitory substances [9–12]. These types of polymicrobial associations have been shown to enhance the resiliency, fitness, and stability of these communities [4, 13, 14]. Maintaining stable oral microbial communities can preserve oral health, thus there is a clear need to identify the interactions between prominent species in host-associated polymicrobial communities and how they might shape not only constituency but physical structure.

To delve into mechanisms underlying taxon-taxon interactions we chose to study the naturally occurring biofilm of human dental plaque. The human supragingival plaque biofilm has been long studied, dating to the very 1^st^ microscopy observations of Antony von Leeuwenhoek in 1683 (ref. [15]). Hypotheses about direct interaction of bacteria and assembly of plaque structures have been developed from work visualizing the spatial structure of plaque, both on extracted teeth [16–19] and on epoxy resin crowns or enamel chips worn in the mouth to allow plaque to develop [16, 20–24]. Recently, more detailed biogeography of plaque structure was observed [25]. One notable association was between *Streptococcus* and other genera at the margins of biofilm structures. Streptococci can influence community interactions due to their ability to rapidly consume carbohydrates, produce large amounts of acidic fermentation products, and excrete antimicrobial substances including reactive oxygen species. These properties can support the growth of some species while excluding others [9, 26–28]. Many oral streptococci including mitis group members are known to produce various antimicrobial substances, including hydrogen peroxide (H_2_O_2_), in aerobic conditions. Therefore, any bacterium adjacent to these *Streptococcus* spp. aerobically would need to have adapted the ability to withstand H_2_O_2_ (refs. [7, 21]). Alongside *Streptococcus* species within the mitis group (*S. mitis, S. oralis, S. australis*, *S. infantis*, and others)*, Haemophilus parainfluenzae* is one of the most abundant and prevalent species in the dental plaque of healthy individuals [29–33]. Whilst frequently characterized as an opportunistic pathogen and implicated in diseases including endocarditis [34], in the oral cavity *H. parainfluenzae* is a commensal and is associated with beneficial immunomodulatory effects [35].

The strains identified as *S. mitis* encompass a broader range of diversity than many bacterial species but appear to be members of a genomically coherent species. Like many species within the mitis group, *S. mitis* strains share less than 95% average nucleotide identity (ANI), the standard threshold used to delineate bacterial species identity [30]. This may be due to the high rate of recombination among some members of the mitis group [36, 37]. The strains identified as *S. mitis* form several distinct clades [38–40]. One clade previously identified as “*S. mitis* biovar 2” has been reclassified as *S. oralis* subsp. *dentisani* [30]. The ANI values indicate the remaining *S. mitis* clades are made up of strains that constitute a continuum of lineages [30]. A more recent analysis indicated that while the remaining clades represent potential subspecies, they are not separate species [40]. For these reasons and because we were primarily interested in *S. mitis* as a representative of the broader mitis group, we elected to treat *S. mitis* as a single species and we selected its type strain as a representative of the species for in vitro experiments.

To identify mechanisms of interaction that take place within this healthy polymicrobial biofilm we investigated interactions between two of its most abundant members, *H. parainfluenzae* and *S. mitis*. Despite the frequent co-occurrence of these species in proximity in vivo, little is known about both the spatial relationship and metabolic mechanisms of interactions between these species. In this study, we observed that these taxa exist in intimate proximity in dental plaque and exhibit localized density dependences that also exist in vitro which are driven by streptococcal H_2_O_2_ production. We also discovered a highly redundant H_2_O_2_ resistance system in *H. parainfluenzae* different from that in other *Haemophilus* species. We then observed multiple mechanisms of *H. parainfluenzae* interaction including NAD auxotrophy complementation by mitis group streptococci and the upregulation of alternative carbon and energy pathways during in vitro coculture that are also observed in in vivo gene expression data. These results provide a robust characterization of *H. parainfluenzae’*s role in the oral microbiota and reveal ways it has evolved to exist alongside streptococci in the oral cavity and likely beyond. This study details interactions between two prominent members of a complex natural biofilm community and allows us to demonstrate mechanisms of interaction that likely help drive micron-scale arrangements between these microorganisms.

## Results

### *H. parainfluenzae* co-occurs with *S. mitis* and related streptococci in human supragingival plaque

**Fig. 1:**
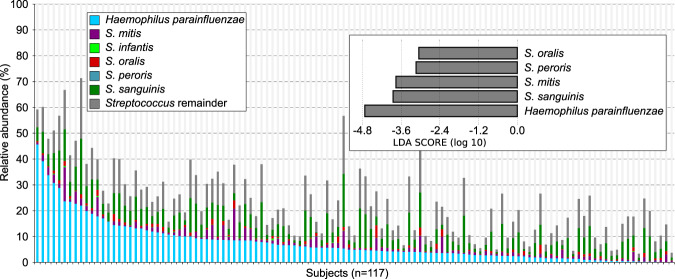
Read abundance data and predicted correlations between taxa in supragingival plaque. Human Microbiome Project (HMP) metagenome data of supragingival plaque was used to plot the relative abundance and prevalence of species of interest including *Haemophilus parainfluenzae* (blue), several mitis group streptococci and all remaining *Streptococcus* spp. (dark gray). (Top Right) The top 25% of subjects based on *H. parainfluenzae* abundance were compared to the remainder via LEfSe analysis. Shown are species enriched in this comparison with an LDA score ≤ −3.0. Full datasets in Table S1 and Fig. S1.

To determine which species *H. parainfluenzae* was most likely to interact with we used microbiome data from 117 subjects sampled by the Human Microbiome Project (HMP). Analysis of HMP species-assigned metagenomic data indicated that *H. parainfluenzae* is an abundant and prevalent member of supragingival plaque detected in all 117 subjects averaging 7.6% relative abundance based on sequence reads (Fig. 1, Table S1). We compared the upper quartile (*n* = 29) of these subjects ranked by highest *H. parainfluenzae* abundance to the remainder of subjects (*n* = 88) via LEfSe analysis [41] to determine which species were significantly likely to co-occur with *H. parainfluenzae* (Fig. 1, Fig. S1). Interestingly, this indicated that individuals enriched in *H. parainfluenzae* have abundant *Streptococcus* sp. especially those in the mitis group. These data were what led us to investigate *H. parainfluenzae* interactions with *S. mitis* as a mitis group representative in order to examine the mechanisms of taxon-taxon interactions.

### Species-specific FISH demonstrates frequent *Haemophilus* spp. co-proximity with a subset of the mitis group and suggests a density-dependent relationship

**Fig. 2:**
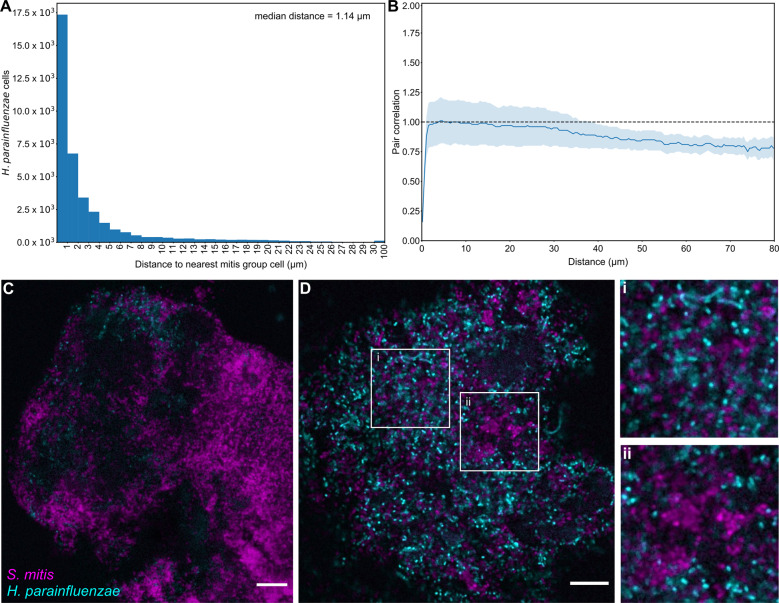
Haemophilus spp. distribution is related to the density of mitis group streptococci in vivo. **A** Histogram of the distance to the nearest *S. mitis* group cell, measured edge-to-edge, from each of the 37,591 *Haemophilus* spp. cells in 41 fields of view. **B** Pair correlations between *Haemophilus* spp. and *S. mitis* group cells. The lighter lines represent the bounds of the 95% confidence interval for the correlation values. The dashed line represents the null hypothesis: the pair correlation equals one. *n* = 41 fields of view. **C** Plaque with sparsely distributed *Haemophilus* spp. (cyan). **D** Plaque with high abundances of both *S. mitis* group (magenta) and *Haemophilus* spp. (i) Most *Haemophilus* spp. cells are within a few microns of the nearest *S. mitis* group cell. Generally, *Haemophilus* spp. cells are randomly distributed with respect to *S. mitis* group. (ii) *Haemophilus* spp. avoids the highest densities of *S. mitis* group. Scale bars indicate 10 µm. Images show hybridization signal from probes Smit651 (mitis group streptococci) and Hpar441 (*Haemophilus* spp.); probe sequences are given in Table S2 and additional probe images are shown in Fig. S2.

To determine whether the large-scale co-occurrence of *H. parainfluenzae* and *S. mitis* was reflected in micrometer-scale spatial structure, we analyzed the organization of these two taxa using fluorescence in situ hybridization (FISH) and imaging. Within the supragingival plaque, *S. mitis* and related streptococci are frequent features of *H. parainfluenzae*’s micron-scale environment. We used FISH probes targeting *H. parainfluenzae* and other *Haemophilus* spp. and *S. mitis* and its close relatives (Fig. S2), referred to hereafter as mitis group species (including *S. infantis*, *S. oralis*, *S. peroris*, and *S. pneumoniae*; see Table S2 for details of probe specificity). Visualizing these species showed that 92% of *Haemophilus* spp. cells in supragingival plaque are located within 10 μm of mitis group species (Fig. 2A, Table S2). The median distance separating a *Haemophilus* spp. cell from the nearest mitis group cell was 1.14 μm. Thus, most cells of *Haemophilus* spp. co-occur with mitis group species at micrometer scales.

In addition to proximity, images suggested a density-dependent relationship between these two taxa, with reduced densities of *Haemophilus* spp. in areas with the highest densities of the mitis group species (Fig. 2C). We quantified this density effect by dividing each image into 1024 blocks of ~6 μm on a side (Fig. 2D) and measuring the density of mitis group species, *Haemophilus* spp., and total bacteria within each block. The mean density of *Haemophilus* spp. increased as the percent of a block covered by the mitis group increased from 0 to around 20% (Fig. S3). This trend is likely due, at least in part, to variation in the overall quantity of plaque in each block because the density of each taxon has a positive linear relationship with the overall plaque density. As the density of the mitis group increased above 20%, the mean *Haemophilus* spp. density decreased, suggesting a density-dependent inhibitory effect of the mitis group on *Haemophilus* spp. growth. Given that both microbiome sequencing and microscopy imaging of in vivo supragingival plaque samples indicate significant co-occurrence and co-proximity between *Haemophilus* spp. and the mitis group (Figs. 1 and 2), it is important to determine the mechanisms that underlie these observations.

### *S. mitis* eliminates *H. parainfluenzae* via production of H_2_O_2_

**Fig. 3:**
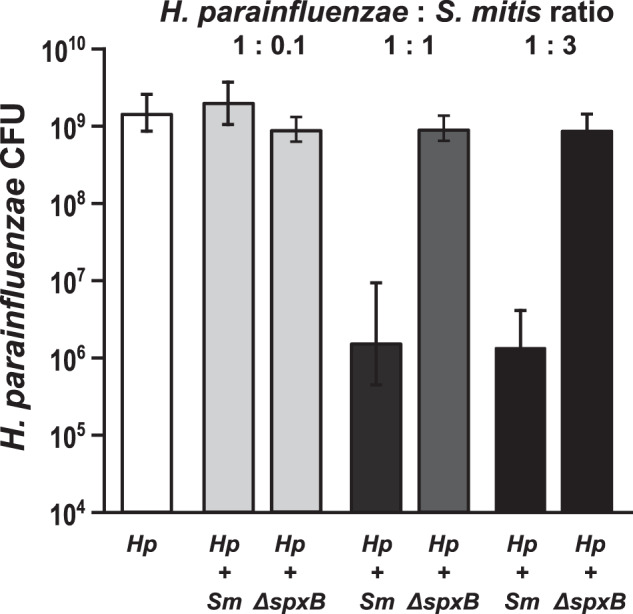
H. parainfluenzae growth is inhibited by Streptococcus mitis produced H2O2 in a dose dependent manner. *H. parainfluenzae* (*Hp*) CFU counts in mono and coculture with wildtype (*Sm*) or a pyruvate oxidase mutant (Δ*spxB*) of *S. mitis. Hp* had an initial inoculum using 10 µL at an OD_600_ of 1, which corresponds to 4.65 × 10^6^ CFU/ml. Wildtype (*Sm*) and *S. mitis* Δ*spxB* with initial inoculums using 10 µL at an OD_600_ of either 0.1, 1 or 3 which corresponds to an average of 2.45 × 10^5^, 1.55 × 10^6^ or 3.45 × 10^6^ CFU/ml. Data are mean CFU counts with error bars indicating standard deviation for *n* ≥ 3. * denotes *p* < 0.001 using a Student’s *t* test compared to monoculture.

Culturing *H. parainfluenzae* and *S. mitis* together under controlled conditions in vitro revealed a dose-dependent interaction: *H. parainfluenzae* was killed by *S. mitis*, but only when abundance of *S. mitis* was high relative to that of *H. parainfluenzae*. We used a colony biofilm model [42, 43] in which polycarbonate membranes were inoculated with *S. mitis* and *H. parainfluenzae*, either separately or together, and permitted to grow for 24 h; cell abundance was then assessed by resuspending the cells and plating for colony counts (see “Methods”). Cocultures inoculated at equal densities revealed that *S. mitis* reduced *H. parainfluenzae* numbers (Fig. 3) nearly 100-fold below inoculum density, indicating active killing. This effect is dose-dependent as we observed a significant reduction in the growth yield of *H. parainfluenzae* compared to monoculture when inoculums of *S. mitis* were either equivalent or threefold greater than *H. parainfluenzae*. However, when *S. mitis* inoculum was tenfold lower than *H. parainfluenzae*, there was no significant change in the growth yield of *H. parainfluenzae* compared to monoculture.

The mechanism of dose-dependent killing of *H. parainfluenzae* involves H_2_O_2_ production by *S. mitis*. The reduction of *H. parainfluenzae* numbers in coculture was abolished if the cocultured strain of *S. mitis* lacked pyruvate oxidase (Δ*spxB*) and thus unable to produce H_2_O_2_. Figure 3 demonstrates that *H. parainfluenzae* growth yield when cocultured with *S. mitis* Δ*spxB* is not significantly different from *H. parainfluenzae* monoculture at any ratio tested, indicating that *S. mitis-*produced H_2_O_2_ is responsible for *H. parainfluenzae* inhibition. Additional coculture experiments indicate that *H. parainfluenzae* was unable to grow in pH neutralized supernatants of *S. mitis* unless they were pre-treated with exogenous catalase (data not shown), further supporting the finding that H_2_O_2_ production limits *H. parainfluenzae* growth in these conditions and that acid production is not sufficient to inhibit *H. parainfluenzae*. Thus, our observed density-dependent exclusion of *H. parainfluenzae* by *S. mitis* in ex vivo samples (Fig. 2) can be explained by H_2_O_2_ toxicity.

Although the density-dependent killing of *H. parainfluenzae* by *S. mitis* implies an antagonistic interaction, the presence of *H. parainfluenzae* enhances *S. mitis* growth yield in biofilm coculture (Fig. S4). This enhancement was abolished when *S. mitis* was cocultured with *H. parainfluenzae* Δ*oxyR* but not with other catalase or cytochrome peroxidase mutants either individually or combined (Fig. S4A). The addition of exogenous catalase elevated *S. mitis* monoculture yields near to those observed in coculture without catalase which suggests that H_2_O_2_ detoxification is primarily responsible for this enhanced yield (Fig. S4B). However, a > 10-fold yield enhancement was still observed in coculture with *H. parainfluenzae* supplemented with exogenous catalase indicating further interactions that benefit *S. mitis* fitness. The role of H_2_O_2_ decomposition in *S. mitis* yield increase was further confirmed when comparing mono vs coculture of *S. mitis* vs its *ΔspxB* mutant where the mutant demonstrated greater yield in monoculture and coculture vs the wild-type with a modest increase coculture yield (Fig. S4C) reminiscent of the wild-type coculture with exogenous catalase.

### Individual H_2_O_2_ sensitive genes do not affect the fitness of *H. parainfluenzae* in coculture with *S. mitis*

The presence of *S. mitis* within a few micrometers of most *H. parainfluenzae* cells in natural plaque suggests that H_2_O_2_ and other inhibitory or promotive compounds excreted by *S. mitis* are reasonably expected to perfuse the substrate in which most *H. parainfluenzae* grow [44]. We investigated the growth effects of known H_2_O_2_ relevant gene products in *H. parainfluenzae* by constructing gene deletions and assessing the fitness of the deletion strains after H_2_O_2_ exposure. Genes in *H. parainfluenzae* relevant to peroxide metabolism include *oxyR* whose gene product is a global transcriptional regulator responsive to H_2_O_2_. Also present is a single catalase (*katA*) essential for H_2_O_2_ resistance in *H. influenzae* [45, 46]. In addition, *H. parainfluenzae* possesses a cytochrome c peroxidase (*ccp*), orthologs of which are important for peroxide resistance in other *Pasteurellaceae* [47], *Campylobacter jejuni* [48], and *H. influenzae* [49]. We also made deletions of the peroxiredoxin (*prx*), peroxiredoxin-glutaredoxin (*pdgX*) and glucose-6-phosphate dehydrogenase (*g6p*) genes known to contribute to oxidative stress protection in *H. influenzae* and other species [46, 50–52].

**Fig. 4:**
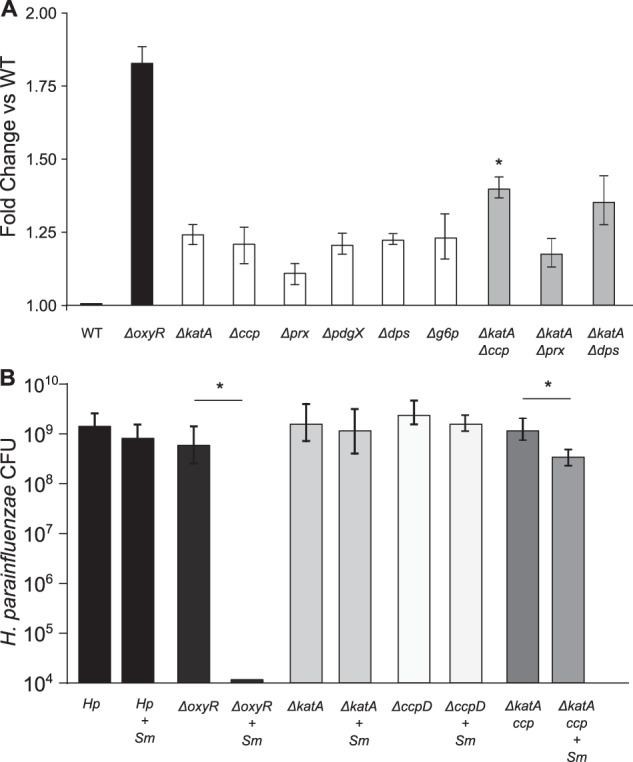
H. parainfluenzae resistance to H2O2 relies on the contribution of multiple genes. We assessed the sensitivity of wildtype *H. parainfluenzae* (WT) to H_2_O_2_ or coculture yield with *S. mitis* vs mutants lacking *oxyR*, catalase (*katA*), cytochrome c peroxidase (*ccp*), peroxiredoxin (*prx*), glutaredoxin-peroxiredoxin (*pdgX*), DNA-binding protein from starved cells (*dps*) and glucose-6-phosphate dehydrogenase (*g6p*). **A** Zones of inhibition by 30% H_2_O_2_ exposure (areas of no visible *H. parainfluenzae* growth) were measured after 24 h and fold change calculated compared to wildtype. Data are the mean fold change relative to WT; error bars indicate standard deviation for *n* ≥ 3. All strains were significantly different from WT, while Δ*katA-*Δ*ccp* was significantly different from Δ*katA* (padj < 0.05), based on *t* test with Bonferroni correction. **B** WT and mutant *H. parainfluenzae* CFU following 24 h coculture with *S. mitis*. *H. parainfluenzae* inoculums were 4.65 × 10^6^ CFU/ml. *S. mitis* inoculum was 2.45 × 10^5^ CFU/ml. Data are represented as mean CFU, error bars indicate standard deviation for *n* ≥ 3. *denotes *p* < 0.05 by Student’s *t* test compared to monoculture.

We quantified changes in H_2_O_2_ resistance by a zone of inhibition assay (Fig. 4A), in which an agar plate inoculated with a lawn of wild-type or deletion-strain *H. parainfluenzae* is exposed to a small filter saturated with H_2_O_2_ and sensitivity is measured as the diameter of the zone of inhibition. As expected, the largest increase in sensitivity was observed in *H. parainfluenzae* with deletions of the global H_2_O_2_-responsive transcriptional regulator Δ*oxyR*, while all other individual gene deletion mutants were less sensitive to H_2_O_2_. A Δ*katA* + Δ*ccp* double mutant demonstrated a significant increase in sensitivity compared to individual mutants, indicating a combinatorial effect of these gene products. Surprisingly, MIC concentrations for many mutants were identical to the wild type (Table S3) indicating that individual contributions of these genes to H_2_O_2_ tolerance are minimal. We next used the colony biofilm model to test the fitness of each mutant in coculture with H_2_O_2_-producing *S. mitis* (Fig. 4B). OxyR was shown to be essential for *H. parainfluenzae* survival in coculture, while deletion of individual genes typically controlled by OxyR showed no significant difference compared to wildtype which contrasts greatly to similar mutants in *H. influenzae* (*katA*) [45, 46] and other species [53–55]. However, the Δ*katA* + Δ*ccp* mutants’ growth yield was significantly inhibited in *S. mitis* coculture again indicating an additive effect of these gene products on H_2_O_2_ resistance. Δ*katA* and Δ*oxyR* mutants were also generated in another oral isolate strain of *H. parainfluenzae* and showed similar trends in regard to H_2_O_2_ sensitivity (Fig. S5). These data suggest that the mechanism of *H. parainfluenzae* resistance to H_2_O_2_ involves a complex multifactorial system unlike other *Haemophilus* spp. yet characterized.

### *S. mitis* and other *Streptococcus* sp. support *H. parainfluenzae* growth

**Fig. 5:**
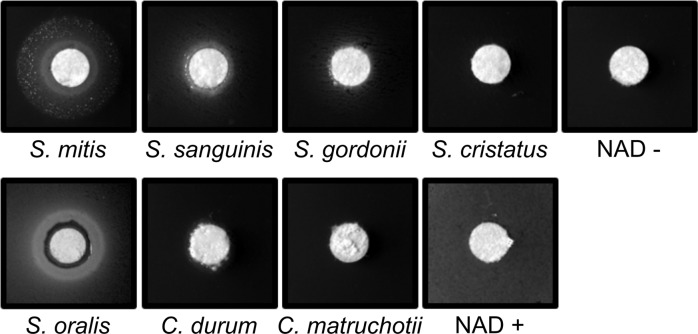
Streptococcus-produced Nicotinamide Adenine Dinucleotide (NAD) supports H. parainfluenzae growth. Cultures of *S. mitis, S. sanguinis, S. gordonii, S. cristatus, Corynebacterium matruchotii* and *C. durum* were grown overnight, normalized based on optical density, and spotted onto paper discs over lawns of *H. parainfluenzae* spread on solid agar medium lacking NAD. Plates were incubated for 48 h before observation. Rings near the disc indicate *H. parainfluenzae* growth. NAD was added a positive control (NAD+).

Like other *Haemophilus sp*., *H. parainfluenzae* is a NAD auxotroph and must obtain NAD, nicotinamide mononucleotide (NMN) or nicotinamide riboside (NR) from the host or other microorganisms [56]. We found that human saliva was unable to support *H. parainfluenzae* growth unless NAD was added (data not shown) suggesting that adjacent microbes are the source of NAD for *H. parainfluenzae* in the oral cavity. As *Corynebacterium* and *Streptococcus* are two of the most abundant genera in supragingival plaque, we tested whether species from these genera could complement NAD auxotrophy. Spot assays on *H. parainfluenzae* lawns on medium lacking NAD showed robust growth of *H. parainfluenzae* adjacent to *S. mitis* and *S. sanguinis* but no other taxa (Fig. 5). These data demonstrate that *H. parainfluenzae* obtains NAD from these taxa when they are in close proximity. It is notable that multiple species that significantly correlate with *H. parainfluenzae* in microbiome data (Fig. 1) also enhance its growth most strongly in the absence of NAD compared to other *Streptococcus* spp. Similar results were also observed in two other oral isolates of *H. parainfluenzae* (Fig. S6).

### In vitro transcriptional responses of *H. parainfluenzae* to *S. mitis*

Transcriptome analysis of *H. parainfluenzae* in aerobic coculture with *S. mitis* indicated a multifaceted response to oxidative stress. We observed that *H. parainfluenzae* significantly differentially expressed 387 genes greater than twofold in coculture (Appendix 1, Fig. S7), compared to monoculture. Surprisingly, the differentially expressed genes did not include catalase; however, based on transcript abundance catalase is well expressed under both conditions. Among genes typically involved in oxidative stress responses, *dps*, which encodes an iron sequestration protein, had a 2.2-fold increase in coculture suggesting that *H. parainfluenzae* is sequestering free intracellular Fe^2+^, to prevent oxidative damage. Surprisingly, however, several other genes involved in H_2_O_2_ were repressed in coculture, including *ccp, pdgX*, thioredoxin (*trxA*), glutaredoxin (*grx*) and thiol peroxidase (*tsa*), demonstrating the complex nature of the oxidative stress response in this species. *H. parainfluenzae* stress responses were induced in coculture, likely due to H_2_O_2_–related damage by *S. mitis*. We observed modest but significant upregulation of the *hfq* chaperone encoding gene, universal stress protein E (*uspE*) and *lexA*. LexA is involved in the repression of genes involved in the SOS response of *E. coli* [56]. Hfq is known to be involved in the stress responses of many species [57]. Paralogs of the Universal stress proteins including *uspE*, are known to be involved in response to DNA damage [58]. Genes likely involved in DNA repair are also induced in coculture including those encoding DNA ligase, exodeoxyribonuclease V beta chain and endonuclease V.

*Streptococcus* spp. are known to rapidly consume carbohydrates, and transcriptional data suggest that *H. parainfluenzae* in coculture switches from carbohydrate consumption to alternative sources of carbon and energy. There was increased expression of genes suggesting the breakdown of glycerophospholipids resulting in the uptake and utilization of glycerol, including the predicted extracellular patatin-like phospholipase (2.5-fold), lysophospholipase L2 (3.8-fold), glycerol uptake facilitator protein (5.3-fold), glycerol kinase (3.4-fold), and a fatty acid degradation regulator (2.1-fold). In addition, there was an increase in expression of fructose 1,6 bisphosphatase (2.8-fold), indicating active gluconeogenesis, consistent with *H. parainfluenzae* growth on 3-carbon intermediates such as glycerol. There was also evidence of the uptake and catabolism of the sialic acid, N-acetylneuraminic acid as suggested by an increase in expression of SHS family sialic acid transporter (twofold), sialic acid utilization regulator (3.6-fold), N-acetylneuraminate lyase (twofold), N-acetylmannosamine kinase (3.8-fold), and N-acetylmannosamine-6-phosphate 2-epimerase (3.1-fold). Last, there was an increase in the expression of genes involved in the oligopeptide transport system, *oppA* (threefold), *oppB* (2.2-fold), *oppC* (2.3-fold), *oppD* (2.7-fold), and *oppF* (2.4-fold). These data together suggest uptake of alternate carbon and energy sources in coculture. *S. mitis* transcriptional responses to *H. parainfluenzae* are the focus of a separate study.

### In vivo transcriptional responses of *H. parainfluenzae* vs in vitro

We hypothesized that in vivo gene expression of *H. parainfluenzae* is largely influenced by *S. mitis* due to their in vivo proximity (Fig. 2) and that our in vitro coculture data may be predictive of *H. parainfluenzae* behavior in vivo. To obtain quantitative data, we compared in vitro *H. parainfluenzae* transcriptomes to two separate in vivo metatranscriptome datasets from healthy dental plaque [59, 60]. We aligned metatranscriptomes to the *H. parainfluenzae* genome generating a dataset of *H. parainfluenzae* transcription within the complex plaque biofilm. We then compared these samples to our in vitro monoculture and then determined which genes differentially expressed in vivo are also expressed in in vitro coculture.

**Fig. 6:**
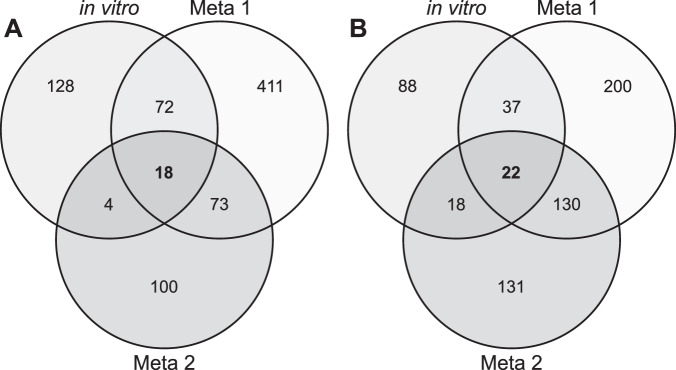
Comparison of coculture gene expression with meta-transcriptome datasets. Mono vs coculture RNASeq results (in vitro) for *H. parainfluenzae* indicated 387 significantly differentially expressed genes (DEG) above twofold. By comparing in vitro monoculture to in vivo metatranscriptome reads from Benítez-Páez 2014 (Meta 1) and Jorth 2014 (Meta 2) we were able to generate two sets of in vivo-specific DEGs to determine differences in (**A**) significantly upregulated or (**B**) significantly downregulated genes shared between in vitro coculture and in vivo conditions.

Comparing significantly differentially expressed gene patterns shared between all 3 datasets (in vitro coculture and both in vivo metatranscriptomes) we observed that 18 genes were mutually upregulated including genes involved in glycerol catabolism, gluconeogenesis, and pili biogenesis (Table S4). We also observed 22 genes that were mutually downregulated including those involved in stringent response, methionine metabolism, and *fur* (Table S5, Fig. 6B). In individual metatranscriptome comparisons, we saw even more genes that were significantly differentially expressed in the presence of *S. mitis* in vitro. This included repression of *ccp*, genes involved in methionine metabolism, stringent response, metal transport genes, *fur*, and induction of genes involved in glycerol catabolism, gluconeogenesis, cell stress, DNA damage/repair related pathways, and peptide/oligopeptide transport (Tables S6-S9).

These data suggest that aspects of *H. parainfluenzae* transcriptional responses in plaque can be recapitulated in in vitro coculture with *S. mitis*. One notably absent overlap between in vitro cocultures vs in vivo datasets was the upregulation of genes involved in lactate oxidation observed in vivo. Since most *Streptococcus* spp. produce lactate as a metabolic end product [26], many oral taxa have evolved the ability to utilize lactate as a carbon source. Metatranscriptome data suggests that *H. parainfluenzae* catabolizes lactate in vivo, which it did not do in coculture indicating that further carbon source competition and crossfeeding is occurring in vivo that did not occur in vitro on complex medium. Overall, these data indicate numerous overlaps of in vitro *H. parainfluenzae* transcriptional responses to *S. mitis* that also occur in vivo where they exist in close proximity.

## Discussion

Our findings reveal multiple factors that may dictate the spatial organization and behavior of two abundant oral commensal bacteria in vivo. The availability and distribution of oxygen has been hypothesized to dictate the spatial organization of bacteria within oral biofilm structures and in mixed communities of soil bacteria, where bacterial respiration creates a reduced environment enabling the growth of strict anaerobes in the biofilm interior [2, 25]. An important facet of oxygen availability is the production of H_2_O_2_ by some microbes, which can be an important mechanism of protection against invading or competing species in a variety of environments from the oral cavity [61] to the surface of the oceans [62]. Also, the exchange of nutrients including electron donors and cofactors or nutrients between taxa can determine growth and interaction between them [8, 63]. Here we demonstrate that streptococcal metabolism can potentially dictate both spatial organization and constituency within a naturally occurring multispecies biofilm.

The production of H_2_O_2_ by early colonizing *Streptococcus* spp. is regarded as an important mechanism of protecting commensals from invading species and is believed to play a role in determining species composition [61]. Our in vitro findings show that *S. mitis* can inhibit or kill *H. parainfluenzae* in a dose-dependent manner via the production of H_2_O_2_. This appears to be mirrored in our in vivo findings where streptococci seemingly exclude *H. parainfluenzae* above a certain density. We assert that this density dependent interaction between these taxa is important for driving heterogeneity and spatial organization within this complex community.

Given the close association in plaque samples between *Haemophilus* spp. and H_2_O_2_ producing streptococci we investigated mechanisms of H_2_O_2_ resistance in *H. parainfluenzae* and demonstrated that in the strains tested it possesses a highly redundant, multifactorial oxidative stress response that substantially differs from other closely related species, including *H. influenzae*. We demonstrated that while loss of OxyR caused a significant increase in H_2_O_2_ sensitivity, loss of catalase or other individual gene products that provide H_2_O_2_ resistance did not (Fig. 4A, Table S3, Fig. S5) which directly contrasts with the importance of catalase in *H. influenzae*, whose deletion leads to its inability to survive high concentrations of H_2_O_2_ (refs. [32, 36]). Streptococcal H_2_O_2_ production is thought to provide a competitive advantage in vivo and presents a source of stress that coexisting bacterial species must tolerate. This ability to deal with streptococci-produced H_2_O_2_ has been demonstrated in multiple species [10, 44, 61, 64]. Given that *H. parainfluenzae* is found closely associated with *S. mitis* in plaque and that they co-occur together in other sites of the oral cavity [65], an intriguing hypothesis is that this redundant system is born from *H. parainfluenzae’s* constant proximity to a H_2_O_2_ producer in multispecies biofilms. Evaluating the regulation of this redundant system is under current investigation by our group. We also noted that the fitness of *S. mitis* in vitro was reduced when cocultured with *H. parainfluenzae* strains impaired in H_2_O_2_ resistance (Fig. S4) and that when exogenous H_2_O_2_ detoxification occurred this fitness was restored (Fig. S4B). However, we also observed that *H. parainfluenzae* still further increased *S. mitis* yield when H_2_O_2_ was not a factor indicating further mutual benefit beyond H_2_O_2_ responses.

Paradoxically, *H. parainfluenzae* can be inhibited by but is found adjacent to *S. mitis*. This could be due to the ability of *S. mitis* to complement the NAD auxotrophy of *H. parainfluenzae*. We observed that abundant plaque *Corynebacterium* spp. were unable to restore *H. parainfluenzae’*s growth, but some oral streptococci and *S. mitis* in particular could (Fig. 5) while host saliva could not. Perhaps this explains why 92% of *H. parainfluenzae* cells lie within 10 µm of *S. mitis* in vivo (Fig. 2A). It is interesting to note that both *H. parainfluenzae* and *S. mitis* are found as commensals not just in the same sites of the human oral cavity [65], but also in other sites in the nasopharynx [66, 67]. Thus, *Streptococcus sp.-*produced NAD could therefore be an important determinant of *H. parainfluenzae’*s ability to survive and colonize various sites of the human body. While this may appear to be disadvantageous to *H. parainfluenzae*, it is also possible given the ubiquitous nature of mitis group streptococci in the oral cavity, that *H. parainfluenzae* evolved this auxotrophy due to the constant availability of extracellular NAD. Interestingly NADP also complements this auxotrophy as *H. parainfluenzae* possesses an NAD kinase that can convert NADP to NAD and vice versa. This kinase was not differentially expressed in our coculture conditions. However, an NADP-specific glutamate hydrogenase was upregulated in coculture 3.1-fold (Supplemental) which suggests that it may play a role in both low pH defense as well as a response to oxidative stress which is under further investigation. These data are reminiscent of other cases in which auxotrophy development confers a fitness advantage demonstrated in broad genome-scale metabolic models [68] as well as direct testing of artificial auxotrophs generated in *E. coli* and *Acinetobacter baylyi* [69] where classes of auxotrophs showed a fitness advantage vs non-auxotrophs when substrates were available. Further, cross-feeding synthetically induced by artificial auxotrophy in *E. coli* conferred a fitness advantage to both strains [70]. Thus, *H. parainfluenzae* may have adapted to exist in close proximity to certain *Streptococcus* spp. due to their NAD/P production whose manner of export is as yet unknown.

When comparing the *H. parainfluenzae* coculture in vitro transcriptome to in vivo supragingival plaque metatranscriptomes (Fig. 6) we observed similar patterns of carbon catabolism including the shift from utilization of sugars to small organic acids and alcohols while downregulating genes in the stringent response suggesting greater access to peptides. Given their in vivo proximity, it is extremely likely that mitis group streptococci are what induce these same *H. parainfluenzae* behaviors in vivo, highlighting a facet of our reductionist approach that can uniquely explain behaviors in a complex multispecies in vivo biofilm. One notable pathway absent from our in vitro cocultures was lactate oxidation which has previously been shown to be critical for co-infection between organisms with *Streptococcus* spp. by cross-feeding on this fermentation product [7]. In vivo, metatranscriptome data indicated that *H. parainfluenzae* was also upregulating lactate oxidation gene products which would be expected in the more diverse, competitive natural plaque environment compared to in vitro coculture alone. *H. parainfluenzae* differentially expressed many other genes in coculture with *S. mitis* that suggest additional stress responses and changes in nutrient availability that have not yet been investigated.

*H. parainfluenzae* and *S. mitis* are found abundantly not just in supragingival plaque but also in other sites of the human oral cavity [33, 65]. The abundance of these two taxa has been associated with health and their reduction is associated with the development of oral squamous cell carcinomas [31, 71]. Both taxa have also been shown to prevent the adhesion and attachment of the oral pathogen *Porphyromonas gingivalis* [72] and their mutual proximity has been observed at the genus level [25]. Therefore, further investigation of the mechanistic interactions that take place between these two species could provide insights into maintaining their presence and thus prevention of disease. Overall, there is a wealth of information and techniques available to study the composition, structure, and gene expression of complex multispecies communities. However, specific mechanisms that exist between individual members of these communities can be hard to discern from broad observational methods. Using a reductionist approach on two highly abundant and prevalent species in the oral cavity we determined how these taxa can support growth of each other and dictate their micron-scale distribution within this environment while suggesting additional mechanisms in use in the greater in vivo community.

## Materials and methods

### Strains and media

Strains and plasmids used in this study are listed in Table S10. Unless indicated, *Streptococcus mitis* was cultured using Brain Heart Infusion (BHI) broth or solid agar supplemented with Yeast extract (YE), *Haemophilus parainfluenzae* had additional supplementation with 15 µg/ml Nicotinamide Adenine Dinucleotide (Sigma-Aldrich) and 15 µg/ml Hemin (Sigma-Aldrich)—(BHI-YE-HP). *Escherichia coli* was grown on Luria Broth (LB). *H. parainfluenzae* and *S. mitis* were grown at 37 °C and 5% CO_2_ for aerobic conditions and in 5% H_2_, 10% CO_2_ and 85% N_2_ in anaerobic conditions. *E. coli* was grown at 37 °C in standard atmospheric conditions with liquid cultures shaken at 200 RPM. Antibiotics were used at the following concentrations: kanamycin 40 µg/ml, vancomycin 5 µg/ml, and spectinomycin 50 µg/ml for *E. coli* and *H. parainfluenzae*. In total, 200 µg/ml spectinomycin was used to select for *H. parainfluenzae* transformants.

### Genomic and plasmid DNA isolation

*H. parainfluenzae* Genomic DNA was isolated using the DNeasy Blood & Tissue kit (Qiagen) according to the manufacturer’s instructions. Plasmid isolations were performed using QIAprep spin miniprep kits (Qiagen).

### Genetic manipulation of *H. parainfluenzae*

Gene deletions were generated using derivatives of a suicide vector pMRKO [7] as listed in Table S10. *H. parainfluenzae* was transformed via conjugation and mutants were screened via PCR and Sanger sequencing. Procedures involved in plasmid construction and *H. parainfluenzae* transformation are outlined in the supplementary methods section (Table S11).

### Read abundance data

MetaPhlAn [73] species-assigned metagenomic sequence data from the Human Microbiome Project (Human Microbiome Project Consortium 2012) for the “Supragingival Plaque” oral site was 1st sorted based on predicted read abundance for *Haemophilus parainfluenzae*. Using the top quartile (highest 25% of samples enriched for *H. parainfluenzae*) we compared these samples to the bottom 75% and performed LEfSe analysis [41] to predict species significantly encountered at higher *H. parainfluenzae* abundance. LDA of a log10 score ≥3 were deemed significant.

### Plaque collection, fixation, and storage

Supragingival plaque was collected according to a protocol approved by New England IRB; all donors provided informed consent. Donors were instructed to refrain from practicing oral hygiene for 24 h before plaque collection. Plaque samples were collected from 7 donors by supervised self-sampling using a toothpick to scrape the supragingival surface of the teeth, avoiding the gingival margin. We fixed the plaque in a solution of 2% paraformaldehyde (PFA) in PBS buffer on ice for 2–6 h. The PFA was removed by three washes with 10 mM Tris HCl buffer (pH 7.5). The samples were stored in a 1:1 (vol/vol) solution of 10 mM Tris HCl (pH 7.5) and 100% ethanol at –20 °C.

### DNA FISH and mounting

Aliquots of plaque were dried on UltraStick Slides (Thermo Scientific) for 10 min at 46 °C. The plaque was hybridized with 2 μM of each probe in a 900 mM NaCl, 20 mM Tris HCl (pH 7.5), 0.01% SDS, and 20% formamide hybridization buffer for 3 h in a humid chamber at 46 °C. Non-hybridized probe was removed by washing the slides in prewarmed 215 mM NaCl, 20 mM Tris HCl (pH 7.5), and 5 mM EDTA wash buffer for 15 min at 48 °C. The slides were rocked once during the wash incubation. The slides were rinsed in chilled deionized water and allowed to mostly air-dry before the samples were mounted in ProLong Gold Antifade mounting medium (ThermoFisher) under a #1.5 coverslip. The slides were dried flat in the dark.

### Imaging

We imaged the hybridized plaque with an LSM 780 Confocal Microscope (Zeiss) with a Plan-Apochromat 40x/1.4 NA objective. Each field of view was simultaneously excited by linear scanning with 405, 488, 561, and 633 nm laser lines. The emission spectra were decomposed by linear unmixing using ZEN software (Zeiss) using reference spectra recorded from pure cultures of reference cells (*Leptotrichia buccalis*) hybridized with the Eub338 probe labeled with the appropriate fluorophore as described above. To obtain a random sample of the masses of plaque large enough to permit spatial analysis, we scanned transects spaced every 5 μm along the coverslip at ×40 magnification and imaged every mass of plaque that was at least 70 μm in diameter and 250 μm away from the previously imaged fields of view. For each donor, we imaged the first 20 fields of view that satisfied these criteria or as many fields of view as were present on the slide. To maximize the number of bacteria captured in each image, we imaged the focal plane closest to the surface of the slide.

### Image analysis

To allow quantitative analysis of the spatial distribution of the taxa of interest, we used FIJI [74] to create binarized images of the subset of *S. mitis* group species, *H. parainfluenzae*, and total bacteria from mitis group, *H. parainfluenzae*, *Streptococcus* spp., *Pasteurellaceae* spp., and Eubacteria probe images as described in the supplementary methods.

We evaluated the pair correlations between *S. mitis* and *H. parainfluenzae* over different distances using a linear dipole analysis performed in Daime 2.2 [75, 76]. For this analysis, the reference space in each image was restricted to the area in the binary bacterial biomass image. We used all possible dipoles with lengths ranging from 0.15 to 99.90 μm in steps of 0.45 μm.

We evaluated trends between the local densities of both taxa, by dividing each field of view into 1024 6.64 μm by 6.64 μm blocks, discarding blocks that did not contain any of the binary bacterial mass image, and calculating the fraction of each block that was covered by the *S. mitis* and *H. parainfluenzae* binary images.

### Mono and coculture assays

Colony biofilm assays were carried out as described previously [43]. Briefly, equal volumes of *H. parainfluenzae* and/or *S. mitis* were spotted either in mono or coculture on sterile 25 mm 0.2 µm polycarbonate membranes (MilliporeSigma) and placed on BHIYE HP agar plates. *H. parainfluenzae* was spotted at an OD_600_ of 1 and *S. mitis* at OD_600_ of either 0.1, 1 or 3. The plates were then incubated for 24 h at 37 °C in 5% CO_2_. The membranes were then transferred to sterile media, mixed to ensure complete resuspension of the colony into the media; serially diluted and plated for CFU enumeration. *S. mitis* was enumerated by counting CFUs on BHI-YE and *H. parainfluenzae* on BHI-YEHP with 5 µg/ml vancomycin.

### Disk diffusion assays

Cultures of *H. parainfluenzae* were grown anaerobically in BHI-YEHP overnight. All strains were then adjusted to an OD_600_ of 1 and 100 µl was spread plated on BHIYE HP plates and incubated aerobically for 2 hours at 37 °C in 5% CO_2_. 5 µl of 30% H_2_O_2_ was then added to a sterile 5 mm paper disk and plates were incubated for 24 hours at 37 °C in 5% CO_2_. The diameters of the zones of inhibition were then measured using a caliper in at least 3 axes.

### Coculture transcriptome sample preparation

RNASeq analyses were carried out on mono and coculture samples following the colony biofilm assays described above. Briefly, *H. parainfluenzae* was spotted on the polycarbonate membranes at an OD_600_ of 1 and *S. mitis* at OD_600_ 0.1. The plates were then incubated for 22 h at 37 °C in 5% CO_2_. The membranes were then transferred onto fresh media for 4 h and immediately placed in RNAlater solution (Ambion). Experiments were carried out in biological duplicates. RNA extraction, library preparation and sequencing were then carried out by the Microbial ‘Omics Core facility at the Broad Institute. Sequences are submitted to the NIH SRA Gene Expression Omnibus (GEO) database and can be found under submission GSE158845.

### Transcriptome analyses

The *H. parainfluenzae* ATCC 33392 genome (GCA_000191405.1) was obtained from NCBI and annotations were generated using RAST under default settings [77–79]. RNASeq reads were aligned, mapped and differentially expressed genes were analyzed using bowtie2 (ref. [80]), HTSeq [81] and DESeq2 (ref. [82]) with a Unix based pipeline, generated in this paper (available at https://github.com/dasithperera-hub/RNASeq-analysis-toolkit). This pipeline was also used to carry out a pathway analysis by mapping DEGs to KEGG orthology (https://www.genome.jp/kegg-bin/get_htext?ko00001). This allowed for improved annotations and the identification of potential pathways that are involved in coculture. The same pipeline was used to analyze *H. parainfluenzae* gene expression in published metatranscriptome datasets [59, 60].

### Complementing nicotinamide adenine dinucleotide (NAD) auxotrophy of *H. parainfluenzae*

Overnight cultures of *H. parainfluenzae* were washed 3 times in 1x Phosphate buffered saline (PBS) and diluted to an OD_600_ of 0.1 and spread plated on a plate containing BHI-YE supplemented with Hemin and 20 units/ml catalase. 5 µL of bacteria at an OD_600_ of 1 was added to a sterile paper disk and incubated for 48 h. Strains used for spotting include *C. matruchotii, C. durum, S. mitis, S. sanguinis, S. cristatus*, and *S. gordonii*.

## Supplementary information

Supplemental Material PDF

Dataset 1

Supplementary Table S1

Supplementary Table S4

Supplementary Table S5

Supplementary Table S6

Supplementary Table S7

Supplementary Table S8

Supplementary Table S9

Supplementary Table S10

Supplementary Table S11
