# Supplementary material for: Mechanisms underlying interactions between two abundant oral commensal bacteria
Source: ISME J. 2021 Nov 3;16(4):948–57. doi: 10.1038/s41396-021-01141-3 (PMC8940909; doi:10.1038/s41396-021-01141-3)
Supplement: Supplementary file 10 — Supplementary Table S10 [file 41396_2021_1141_MOESM10_ESM.pdf]

Table S1: Full species assigned sequence abundance dataset used in Figure 1.

Table S2: DNA FISH probes

Probe	Fluorophore	Target Taxon	Probe Sequence 5'-3'	Reference
Eub338	At655 5' and 3'	Bacteria (domain)	GCTGCCTCCCGTAGGAGT	Amann et al. 1990
Pas111	Dy615 5' and 3'	Pasteurallaceae (family)	TCCCAAGCATTACTCACC	Valm et al. 2011
Str405	RRX 5'	Streptococcus (genus)	TAGCCGTCCCTTTCTGGT	Paster et al. 1998
Smit651	Dy415 5' and 3'	*S. mitis, S. infantis, S. oralis, S. peroris, S. pneumoniae, S.australis, S. lactarius, S. sp. HMT 061, 064, 074, 423*	CCCCTCTTGCACTCAA	Wilbert et al. 2020
Hpar441	Dy490 5' and 3'	*H. parainfluenzae, H. pittmaniae, H. sputorum*	ACTAAATGCCTTCCTCGCTAC	this paper

Table S3: Minimum inhibitory concentrations in BHI-YE HP

Strain	MIC (μM)
Wildtype	834
Δ*katA*	834
Δ*ccp*	834
Δ*oxyR*	104

Table S4: *H. parainfluenzae* genes induced in *in vitro* coculture and both *in vivo* metatranscriptomes

Table S5: *H. parainfluenzae* genes repressed in *in vitro* coculture and both *in vivo* metatranscriptomes

Table S6: *H. parainfluenzae* genes upregulated in *in vitro* coculture and *in vivo* metatranscriptome Benítez-Páez et al., (2014)

Table S7: *H. parainfluenzae* genes downregulated in *in vitro* coculture and *in vivo* metatranscriptome Benítez-Páez et al., (2014)

Table S8: *H. parainfluenzae* genes induced in *in vitro* coculture and *in vivo* metatranscriptome Jorth et al., (2014)

Table S9: *H. parainfluenzae* genes repressed in *in vitro* coculture and *in vivo* metatranscriptome Jorth et al., (2014)

Table S10: Strains and plasmids used in this study

Table S11: Primers used in this study

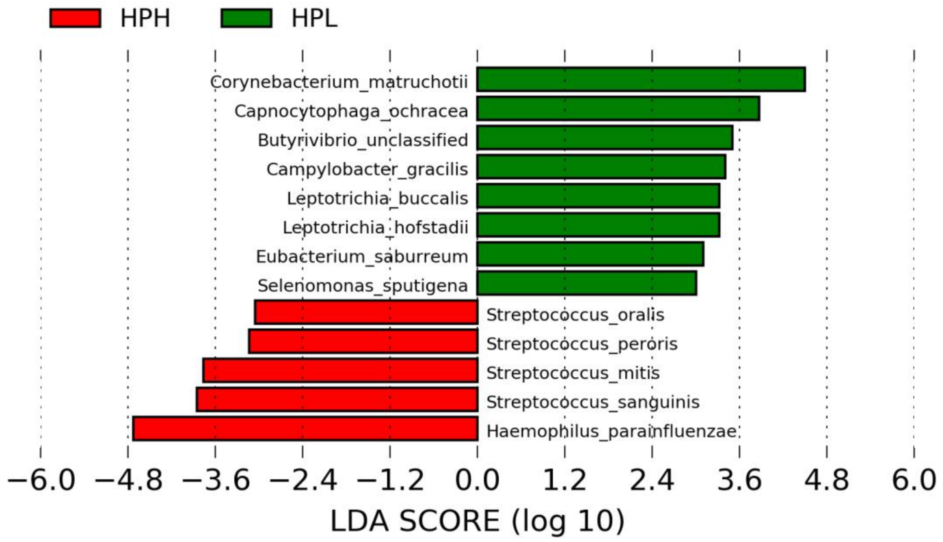

Figure S1: Full LEfSe plot data from Figure 1 indicating most and least enriched organisms based on the top quartile of *H. parainfluenzae* total abundance across subjects. Shown are significant LDA scores ≥ 3.0 or ≤ -3.0.

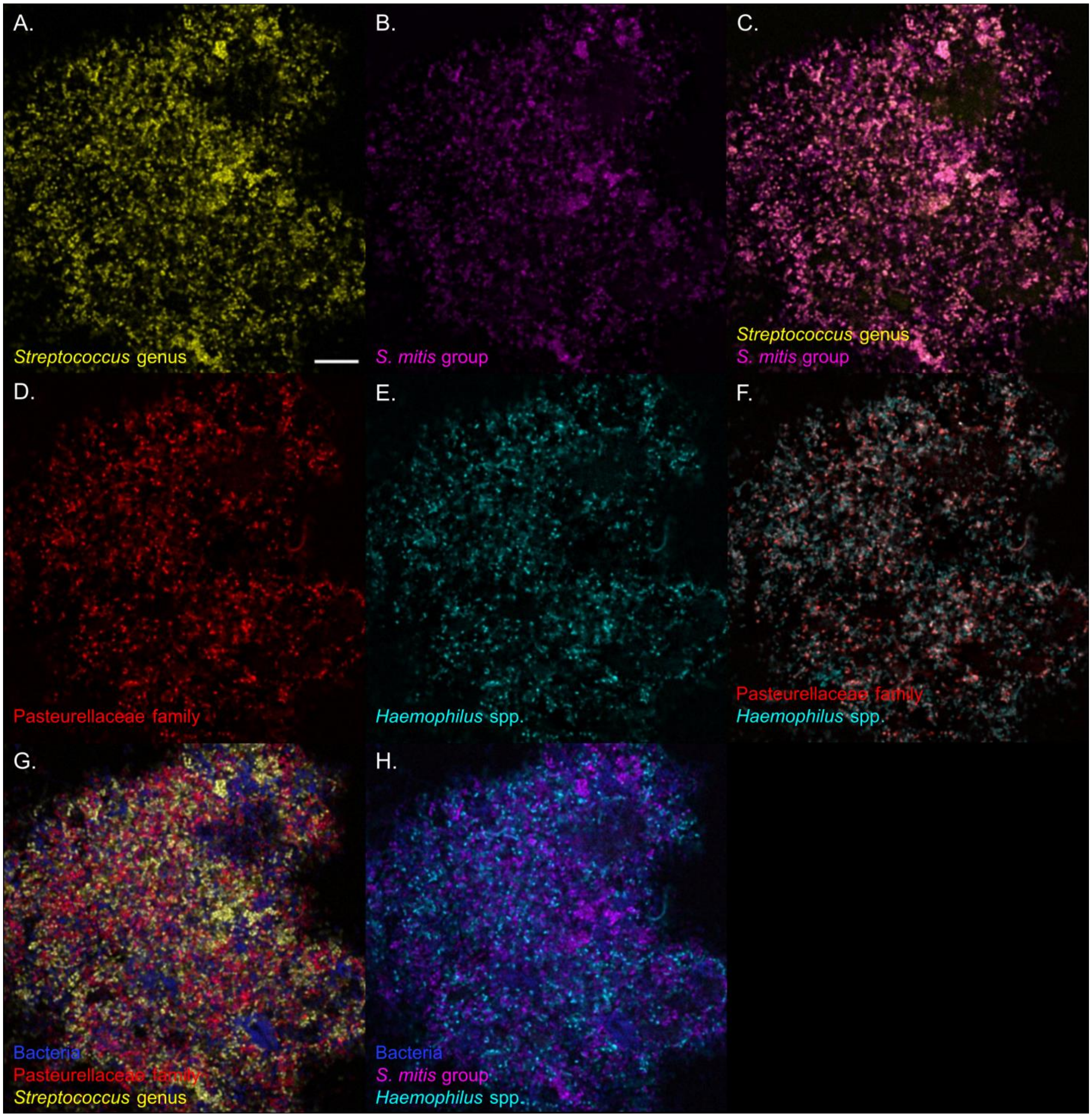

Figure S2: All fluorescence probe images used in the *ex vivo* spatial analysis are shown for the field of view from Fig. 2D. Fluorescence images are shown for the following probes: Str405 targeting genus *Streptococcus* (A), Smit651 targeting *S. mitis* group (B), merged *Streptococcus* and *S. mitis* group (C), Pas111 targeting family *Pasteurellaceae* (D), Hpar441 targeting *H. parainfluenzae* (E), merged *Pasteurellaceae* and *H. parainfluenzae* (F), merged Eubacteria (Eub338), Streptococcus, and Pasteurellacae (G), and merged *S. mitis* group and *H. parainfluenzae* (H). The scale bar in (A) represents 10 μm. The key indicates the color corresponding to each probe. Only cells appearing in both the species-specific image and the genus- or family-specific image were considered positively identified and used for quantitative image analysis.

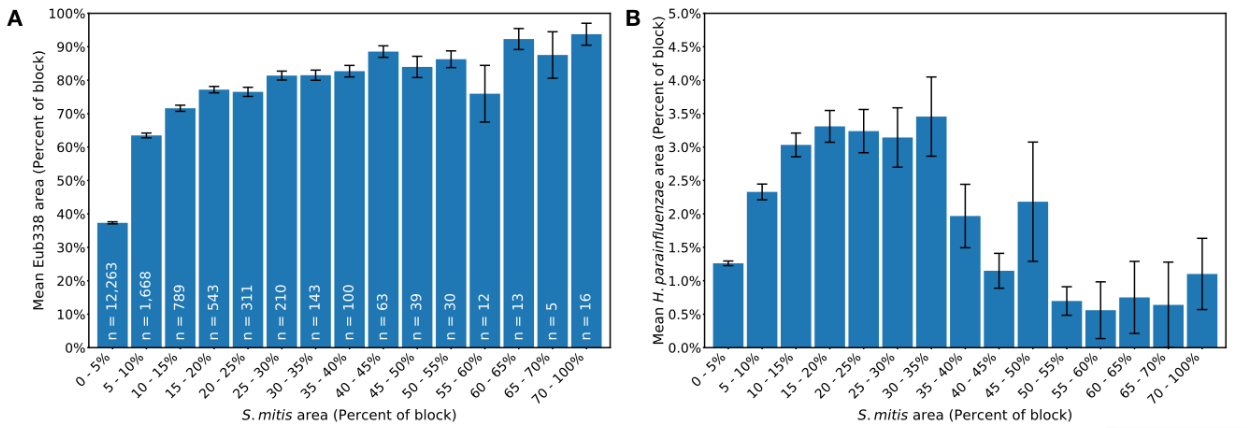

Figure S3: Total bacteria and *H. parainfluenzae* density relative to *S. mitis*. Bar heights represent the mean densities of (A) Eub338-labeled bacteria and (B) *H. parainfluenzae* with respect to the mean *S. mitis* densities for 16,205 blocks of 6.64 μm by 6.64 μm, from 41 fields of view. The number of blocks for each range of *S. mitis* densities is shown in S1A. The error bars represent ± 1 standard error.

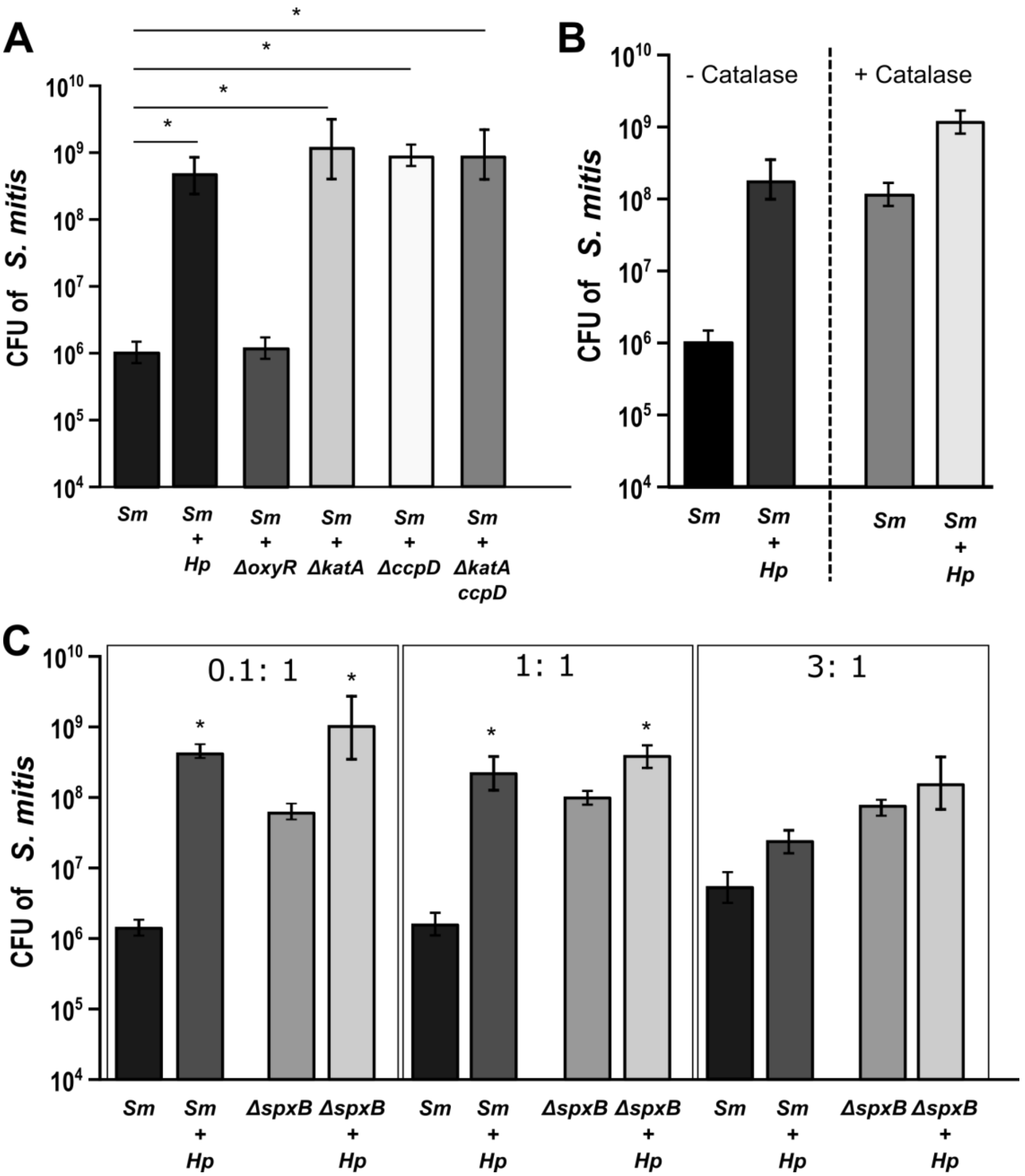

Figure S4: *H. parainfluenzae* H_2O_2 detoxification aids *S. mitis* growth. (A) *S. mitis* CFU when cocultured with *H. parainfluenzae* WT and indicated H_2O_2 resistance gene deletion mutants. Data are mean CFU, error bars indicate standard deviation for n=3. *denotes $p < 0.001$ by Student's t-test compared to monoculture CFU. (B) *S. mitis* CFU in mono and coculture with the addition of 20U/ml of exogenous catalase following incubation for 24 hours. (C) CFU counts of *S. mitis* (*Sm*) and the pyruvate oxidase mutant of *S. mitis* (*ΔspxB*) in mono and coculture with wildtype *H. parainfluenzae* (*Hp*). Hp had an initial inoculum of 4.65 x 106 CFU/ml. Wildtype (*Sm*) and *S. mitis ΔspxB* with initial inoculums of 2.45×10^5, 1.55×10^6 or 3.45×10^6 CFU/ml. Data are mean CFU and error bars indicate standard deviation for n≥3. *denotes $p < 0.05$ by Student's t-test compared to monoculture.

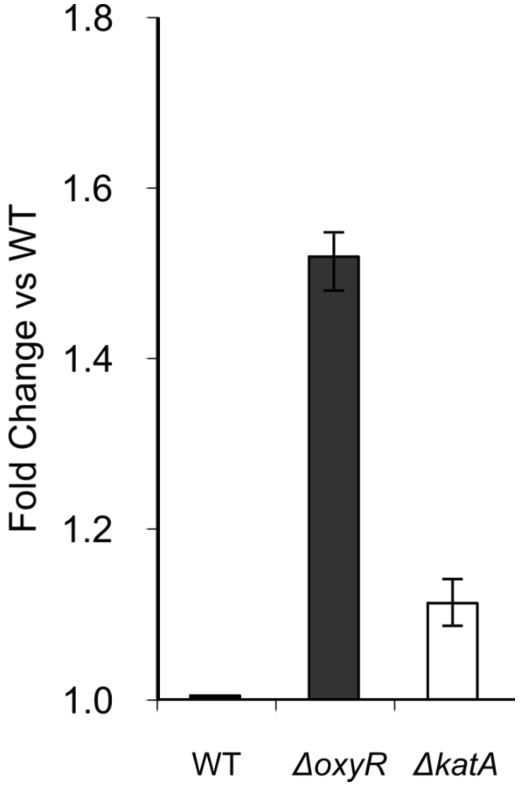

Figure S5. ***H. parainfluenzae*** **resistance to H_2O_2 for indicated mutants.** Deletion mutants of *oxyR* and catalase (*katA*) were generated in *H. parainfluenzae* oral isolate strain MR0305 and yielded similar phenotypes to identical mutants in strain ATCC33392 (Figure 4). All strains were significantly different from the WT, $p < 0.05$ by Student's t-test. Data are the mean fold change relative to WT; error bars indicate standard deviation for $n \geq 3$.

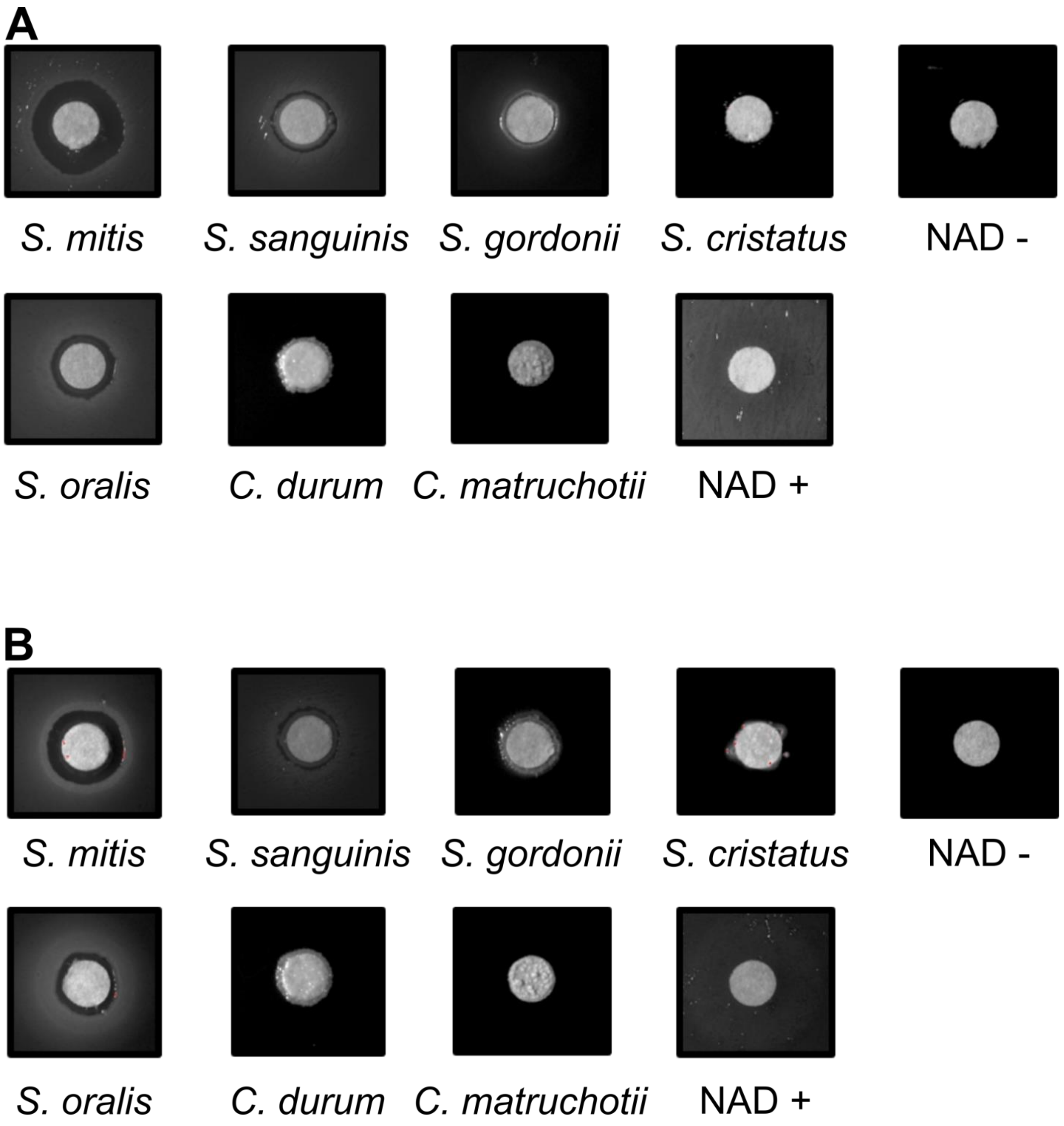

Figure S6. *Streptococcus*-produced Nicotinamide Adenine Dinucleotide (NAD) supports *H. parainfluenzae* growth in multiple strains. Cultures of indicated species were grown overnight, normalized based on optical density, and spotted onto paper discs over (A) Lawns of *H. parainfluenzae* strain MR0206 or (B) lawns of *H. parainfluenzae* strain MR0305 spread on solid agar medium lacking NAD. Plates were incubated for 48 h before observation. Rings near the disc indicate H. parainfluenzae growth. NAD was added a positive control (NAD+).

A

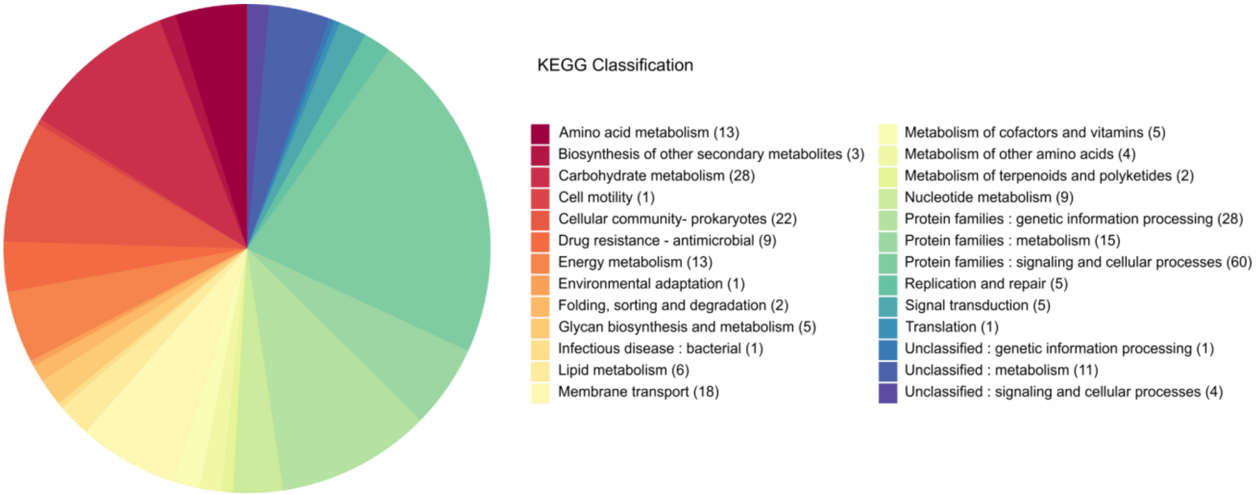

B

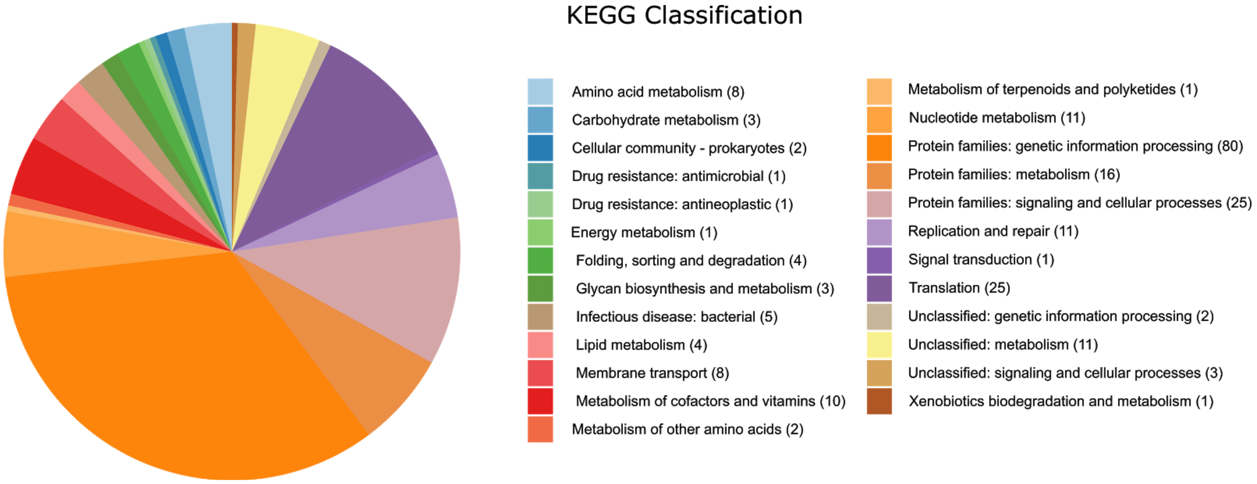

Figure S7: *H. parainfluenzae* transcriptional response to *S. mitis*. (A) Pathways induced in *H. parainfluenzae* when cocultured with *S. mitis* based on number of genes that have > 2 fold increase in expression. (**B)** Pathways repressed when *H. parainfluenzae* is cocultured with *S. mitis* based on a 2 fold decrease in coculture.

Supplementary Methods

Gene deletion in *H. parainfluenzae*

1kb flanking regions of the target gene were amplified via PCR using Q5 High-fidelity 2X Mastermix and primers indicated in Table S9. For allelic exchange, these fragments were assembled to flank a kanamycin resistance cassette via isothermal assembly using the NEBuilder HiFi DNA Assembly MasterMix (New England Biolabs) and cloned into pMRKO. This resulting reaction was then transformed into NEB5α competent cells using the manufacturer's instructions (New England Biolabs). Plasmid constructs were verified via restriction digests and Sanger sequencing. After screening, plasmids were transformed into the donor strain MFD-pir, using the TSS transformation method (6).

These strains were then used to conjugate into *H. parainfluenzae*. Briefly, washed cells of *H. parainfluenzae* overnight cultures were subjected to heat-shock (46°C for 6 minutes) and combined with the donor strain by spread plating on a BHI-YE HP agar plate supplemented with 0.3 mM di-amino pimelate (DAP). Plates were then incubated overnight at 37°C in 5% CO_2. Cells were then harvested and dilutions were plated on BHI-YE HP with 40 µg/ml of kanamycin and incubated for 24-48 hours at 37°C in 5% CO_2. Mutants were then screened by testing for sensitivity to spectinomycin (spectinomycin resistance cassette on pMRKO backbone), PCR and Sanger sequencing.

The markerless deletion of genes in *H.parainfluenzae* involved modifications to the above protocol. 1kb flanking regions were amplified and cloned into a pMRKO derivative containing a *sacB* gene (pEAKO - Table S8). Plasmids were then transformed into *H. parainfluenzae* via conjugation as described above. After transformation, cells were subjected to counterselection by plating on BHI-YE HP containing 10% sucrose for 4-5 days. Mutants were then screened via PCR and Sanger sequencing.

Image Analysis

To allow quantitative analysis of the spatial distribution of the taxa of interest, we used FIJI to create binarized *S. mitis*, *H. parainfluenzae,* and bacterial mass images. A slight misalignment of the Smit651 channel was brought into closer alignment with the other channels by shifting it up by 2 pixels, cropping 3 pixels off each edge, and re-scaling the channel image to regain the original 2,048 by 2,048 resolution. The noise in each channel was reduced by applying a median filter with a radius of 3 pixels. To create a bacterial biomass mask, the Eub338 channel was automatically segmented by thresholding with the global Otsu method (7) and dilating the segmented area by 3 pixels. The *S. miti*s channel was created by segmenting the Str405 and Smit651 channels with the local Bernsen and global RenyiEntropy automatic thresholding methods, respectively (8,9). Both segmented images were combined using the Boolean "AND" operator to retain the pixels appearing in both images. The *H. parainfluenzae* channel was created by segmenting the Pas111 and Hpar441 channels with the local Bernsen and global RenyiEntropy automatic thresholding methods, respectively. Both segmented images were combined using the Boolean "AND" operator. To ensure that there was a sufficiently large area of *H. parainfluenzae* in the images for reliable analysis, only the 41 fields of view in which at least 1% of the bacteria mass was covered by *H. parainfluenzae* in the associated binary image were used for the subsequent analyses.

References

1. Ramsey MM, Rumbaugh KP, Whiteley M. Metabolite cross-feeding enhances virulence in a model polymicrobial infection. PLoS Pathog. 2011 Mar;7(3):e1002012.

2. Ferrières L, Hémery G, Nham T, Guérout A-M, Mazel D, Beloin C, et al. Silent mischief: bacteriophage Mu insertions contaminate products of Escherichia coli random mutagenesis performed using suicidal transposon delivery plasmids mobilized by broad-host-range RP4 conjugative machinery. J Bacteriol. 2010 Dec;192(24):6418–27.

3. Redanz S, Treerat P, Mu R, Redanz U, Zou Z, Koley D, et al. Pyruvate secretion by oral streptococci modulates hydrogen peroxide dependent antagonism. ISME J. 2020 May;14(5):1074–88.

4. Narayanan AM, Ramsey MM, Stacy A, Whiteley M. Defining Genetic Fitness Determinants and Creating Genomic Resources for an Oral Pathogen. Appl Environ Microbiol. 2017 Jul 15;83(14).

5. Schäfer A, Tauch A, Jäger W, Kalinowski J, Thierbach G, Pühler A. Small mobilizable multi-purpose cloning vectors derived from the Escherichia coli plasmids pK18 and pK19: selection of defined deletions in the chromosome of Corynebacterium glutamicum. Gene. 1994 Jul 22;145(1):69–73.

6. Chung CT, Niemela SL, Miller RH. One-step preparation of competent Escherichia coli: transformation and storage of bacterial cells in the same solution. Proc Natl Acad Sci U S A. 1989 Apr;86(7):2172–5.

7. Otsu N. A Threshold Selection Method from Gray-Level Histograms. IEEE Trans Syst Man Cybern. 1979 Jan;9(1):62–6.

8. Bernsen, John. Dynamic Thresholding of Grey-Level Images. 8th Int Conf Pattern Recognit Paris Fr. 1986 Aug;1251–5.

9. Kapur JN, Sahoo PK, Wong AKC. A new method for gray-level picture thresholding using the entropy of the histogram. Comput Vis Graph Image Process. 1985 Mar;29(3):273–85.

10. Amann RI, Krumholz L, Stahl DA. Fluorescent-oligonucleotide probing of whole cells for determinative, phylogenetic, and environmental studies in microbiology. J Bacteriol. 1990 Feb;172(2):762–70.

11. Valm AM, Mark Welch JL, Rieken CW, Hasegawa Y, Sogin ML, Oldenbourg R, et al. Systems-level analysis of microbial community organization through combinatorial labeling and spectral imaging. Proc Natl Acad Sci U S A. 2011 Mar 8;108(10):4152–7.

12. Paster BJ, Bartoszyk IM, Dewhirst FE. Identification of oral streptococci using PCR-based, reverse-capture, checkerboard hybridization. Methods Cell Sci. 1998 Mar 1;20(1):223–31.

13. Wilbert SA, Mark Welch JL, Borisy GG. Spatial Ecology of the Human Tongue Dorsum Microbiome. Cell Rep. 2020 Mar 24;30(12):4003-4015.e3.
